# Radiation and Anti-Cancer Vaccines: A Winning Combination

**DOI:** 10.3390/vaccines6010009

**Published:** 2018-01-30

**Authors:** Alexandra Cadena, Taylor R. Cushman, Clark Anderson, Hampartsoum B. Barsoumian, James W. Welsh, Maria Angelica Cortez

**Affiliations:** 1 Department of Experimental Radiation Oncology, The University of Texas MD Anderson Cancer Center, Houston, TX 77030, USA; APCadena@mdanderson.org (A.C.); TRCushman@mdanderson.org (T.R.C.); 2 Paul L. Foster School of Medicine, Texas Tech University Health Sciences Center El Paso, El Paso, TX 79415, USA; clark.anderson@ttuhsc.edu; 3 Department of Radiation Oncology, The University of Texas MD Anderson Cancer Center, Houston, TX 77030, USA; HBarsoumian@mdanderson.org (H.B.B.); jwelsh@mdanderson.org (J.W.W.)

**Keywords:** radiotherapy, in situ vaccine, cancer, immunotherapy, viral vaccines, protein/peptide vaccines, toll-like receptors

## Abstract

The emerging combination of radiation therapy with vaccines is a promising new treatment plan in the fight against cancer. While many cancer vaccines such as MUC1, p53 CpG oligodeoxynucleotide, and SOX2 may be great candidates for antitumor vaccination, there still remain many investigations to be done into possible vaccine combinations. One fruitful partnership that has emerged are anti-tumor vaccines in combination with radiation. Radiation therapy was previously thought to be only a tool for directly or indirectly damaging DNA and therefore causing cancer cell death. Now, with much preclinical and clinical data, radiation has taken on the role of an in situ vaccine. With both cancer vaccines and radiation at our disposal, more and more studies are looking to combining vaccine types such as toll-like receptors, viral components, dendritic-cell-based, and subunit vaccines with radiation. While the outcomes of these combinatory efforts are promising, there is still much work to be covered. This review sheds light on the current state of affairs in cancer vaccines and how radiation will bring its story into the future.

## 1. Introduction 

Today, not only do we know much more about the immune system’s role in fighting infectious diseases, but we have also made great progress in uncovering how the immune system fights tumors. As we gear towards an era of so-called “personalized medicine” in the treatment of cancer, this review looks to the future of vaccination and the role that radiotherapy (XRT) and anti-cancer vaccines play in priming and sustaining a patient’s anti-cancer immunity. While history has largely placed radiation therapy in the realm of localized disease control, current evidence sheds light on radiation’s ability to induce systemic immune responses [1]. The fastest growing field in cancer research is that of immuno-oncology; thus, this review emphasizes the new cancer vaccine therapies that are in development as well as the role vaccines play in creating anti-cancer immunity in combination with radiation.

## 2. Cancer Vaccines 

Cancer vaccines are vaccines that induce an immune response specific to a type of cancer in order to treat or prevent the development of that cancer. They may be categorized into tumor-associated antigen-based vaccines and dendritic-cell-based vaccines. Tumor-associated antigen-based vaccines contain a tumor-specific antigen for activation of immune cells. Dendritic-cell-based vaccines are aimed at promoting antigen presentation by dendritic cells to induce antitumor responses. To date, Sipuleucel-T (a dendritic-cell-based vaccine), for advanced prostate cancer, is the only cancer vaccine that has shown effectiveness compared to placebo in a Phase III clinical study and has been approved by the FDA for use in cancer patients [2]. There is vast potential for anti-neoplastic vaccines in cancer patients, and many studies are testing their effectiveness in a variety of cancers, from HER2 vaccines in breast and bladder cancer (NCT01730118) to MAGE-3 (NCT00290355), p53 (NCT00049218), and MUC1 (NCT02140996, NCT00415818, NCT03300817) vaccines in lung cancers and many more. However, still more research is required to realize the potential of these vaccines as solo or combination therapies. Highlighted below are some of the well-studied and promising anti-cancer vaccine targets.

Mucin 1 (MUC1) is a cell membrane glycoprotein highly expressed in many cancers including non-small cell lung cancer (NSCLC), breast, colorectal, prostate, pancreatic, ovarian, and multiple myeloma [3]. In contrast to low-level luminal or apical expression of the heavily glycosylated MUC1 on normal colonic epithelial cells, cancer cells express high levels of the hypoglycosylated form of MUC1. Researchers have developed a vaccine containing a synthetic MUC1 peptide, to be injected in to patients with premalignant colon adenomas [4]. The vaccine showed immunogenicity in almost half of patients. Those in whom no immunogenic response was elicited had higher levels of circulating myeloid-derived suppressor cells (MDSCs) [3]. MDSCs are known to be important in mediating the immunosuppressive tumor micro-environment [5] and regulate facets of the immune system even in a healthy individual [6]. The authors suggest there may be a premalignant immunosuppressive state, characterized by increased MDSCs, which prevented patients from benefitting from the vaccine [4]. There was no difference in T regulatory cells (Tregs) between responders and non-responders. The BLP25 lipopeptide vaccine, a 25-amino acid MUC1 sequence is being tested in unresectable NSCLC. The vaccine led to increased survival in NSCLC patients in a Phase II clinical trial; however, no assumptions correlating immune response with survival could be produced from the study [7].

Tumor Protein P53 (TP53, also known as p53) accumulates in great numbers in some cancers and offers a targetable cancer antigen [8,9]. A p53 vaccine has been tested in patients with small cell lung cancer (SCLC), head and neck cancer, and ovarian cancer [10,11,12,13]. While not shown to generate an antitumor response, a p53-modified adenovirus-transduced dendritic cell vaccine was found to be safe and appeared to sensitize tumor cells to subsequent chemotherapy [10,11]. It may be the case that chemotherapy allows activated immune cells to detect and kill tumor cells more easily by downregulating tumor immunosuppressive factors or upregulating p53. The mechanisms behind this synergism require further research. In a Phase I trial, 69% of patients with head and neck squamous cell carcinoma had an immunogenic response to p53 vaccination and Treg frequencies were consistently decreased [13]. A Phase II trial investigated a p53 vaccine in patients with recurrent ovarian adenocarcinoma and found that it induced a T-cell response but did not improve clinical outcomes [12].

The embryonic stem cell gene SRY (sex determining region Y)-box 2 (*SOX2*) is an oncogenic driver in NSCLC and may be a good candidate for antitumor vaccination. In mice, a SOX2 vaccine inhibited the growth of the TC-1 lung cancer cell line characterized by high SOX2 production [14]. Studies suggest that both humoral immune responses [15] and T-cell responses [16] against SOX2 may correlate with clinical response in patients receiving immunotherapy. Many NSCLC patients mount a T-cell response against SOX2 naturally. In these patients, programmed cell death-1 (PD-1) blockade resulted in tumor regression as well as the amplification of SOX2-specific immune responses [17].

Cancer vaccines have already shown great promise, but many questions remain regarding vaccination as an anti-neoplastic therapy. There are many ongoing studies investigating their effectiveness as solo treatment, but also in combination with chemotherapies, radiation, immunotherapy, and other interventions. Further research should also be aimed at the role of vaccination in preventing cancer.

## 3. Radiation as an In Situ Vaccine

Previously, radiation therapy was believed to eliminate cancer solely through direct and indirect damage to DNA causing cell death. However, the occasional observation of systemic response to local treatment with radiation therapy, known as the abscopal effect, has caused researchers to reevaluate this theory. Abscopal phenomena have been documented in many different solid tumor types, including melanoma, NSCLC, renal cell carcinoma, hepatocellular carcinoma, and more [18]. In 1979, Helen Stone demonstrated the importance of intact host immunity to have a response to radiation therapy, clearly demonstrating, for the first time, the critical relationship between the immune system and radiation therapy [19]. It is now hypothesized that radiation therapy can elicit a tumor-specific immune response that not only targets cancer cells locally but can also travel to distant sites of disease and act as an in situ vaccine, resulting in a systemic response [20,21]. Additional evidence for this theory is supported by the synergistic effects of radiation and immunotherapies, which have demonstrated improved clinical response, disease free survival, overall survival, and time to recurrence in multiple cancer histologies [22,23,24].

In 2004, Demaria et al. demonstrated that, in a preclinical mammary carcinoma murine model, combining radiation with Flt3-L, a growth factor, led to an abscopal response while neither therapy alone elicited any control of the abscopal tumor [20]. To verify that the abscopal response was a tumor-specific response, researchers inoculated one group of mice with two mammary carcinoma tumor sites and one lymphoma site. The abscopal response was only seen in the abscopal mammary carcinoma and not at the lymphoma site, cleverly exemplifying the antitumor-specific immunity generated by irradiation of one tumor site. Additional preclinical evidence emerged in 2005 when Lugade and colleagues sought to explore the idea of radiation serving as an in situ vaccine [25]. The researchers irradiated murine melanoma cells and found that, after radiation, mice had increased numbers of antigen-presenting cells and increased numbers of interferon-γ-secreting T cells. Lugade et al. also infused fluorescent-labeled ovalbumin-specific cells and CD8^+^ T cells into C57BL/6 mice bearing B16/OVA tumors. One group received no radiation, another group received 3 Gy, and the third group received 15 Gy. Tumors were analyzed 24 or 72 h after infusion. Mice with tumors that had received radiation had significantly higher tumor antigen-specific T cells at both time points. Current preclinical research is now beginning to uncover specific molecular and cellular mechanisms by which radiation primes the immune system. Our group recently demonstrated the ability of radiation to resensitize NSCLC tumors resistant to anti-PD-1 via increased secretion of type I interferons, leading to increased expression of MHC class I [26].

Although there is much promise in utilizing radiotherapy as a powerful immunogenic stimulus, research has also shown that XRT has the ability to decrease immunogenic response through several pathways. In 2015, Vanpouille-Box and colleagues demonstrated the undesirable effect of XRT to upregulate TGF-β [27], which is known to increase immunosuppressive CD4^+^ T regulatory cells, polarization of M1 to M2 macrophages, and inhibition of dendritic cell maturation. Next, they highlighted the ability of TGF-β blockade in addition to XRT to increase CD8^+^ T-cell antitumor response. Other effects of XRT, such as increased CSF1, HIF-1α, and accumulation of adenosine, have demonstrated undesirable immunosuppressive effects [28,29,30]. Targeting these effects may further potentiate the synergism already seen between XRT and immunotherapy.

In addition to preclinical data, numerous clinical observations have been published supporting the concept of radiation serving as an in situ vaccine [31,32,33]. With the relationship between the immune system and radiation therapy being firmly established, it is no surprise that there are dozens of clinical trials underway investigating the benefits of combining radiation therapy with immunotherapies. Critical to progress in the field of oncology, such trials must analyze the immunologic landscape before, during, and after treatments. In doing so, we gain insight into the molecular and cellular mechanisms contributing to radiation therapy’s generation of systemic tumor-specific immune responses.

## 4. TLR Vaccines

Another vital candidate in the arena of cancer vaccines are toll-like receptor (TLR) agonists, which are known to bridge innate and adaptive immunities through activation, cytokine release, and interferon production by dendritic cells (DCs) and macrophages. Combining radiation with TLR stimulation helps increase the availability and the presentation of tumor-associated antigens (TAAs), respectively. With that in mind, several preclinical studies were conducted utilizing TLR agonists in combination with radiation. One particular group treated mice affected by B-cell lymphoma (A20 cell line) and T-cell lymphoma (EL-4 cell line) with R848, a TLR-7 agonist, plus radiation therapy [34]. Along with radiation the agonist was administered five times a week. Results showed a significant tumor regression compared to when either treatment strategy was used alone. Dovedi et al. posited that the expansion of CD8^+^ T cells by the combination of TLR-7 agonist with radiation led to tumor regression [34]. There have been numerous pre-clinical and clinical studies on the TLR-7 agonist, imiquimod. Adams et al. have shown that imiquimod can cause an immune-mediated rejection of skin metastases of breast cancer patients [35]. Ten patients were enrolled, with two patients receiving a partial response and evidence that the TLR-7 agonist in question helped locally produce cytokines and tumor lymphocytic infiltrate, which in turn created a pro-immunogenic microenvironment [35]. In a different study with a TLR-4 agonist, researchers showed a direct correlation between radiation dose and upregulation of TLR-4-pathway-associated molecules such as CD14, MD2, and MyD88, culminating in the production of IL-12 and IL-18 cytokines by murine macrophages [36].

CpG oligodeoxynucleotide is a toll-like receptor 9 agonist used to treat many cancers through stimulating the innate immune system [37,38]. Phase I and II trials in glioblastoma, mantle cell lymphoma, Wilms tumor, and melanoma have demonstrated safety, but little evidence that these treatments will improve survival without complimentary therapy (NCT02680184, NCT00490529), [38,39,40,41]. Numerous studies have been done with CpG-B vaccine and melanoma patients. Molenkamp et al. studied the effect of preoperative local administering of CpG-B vaccine on the T-cell and dendritic cell populations of Stage 2 and Stage 3 melanoma patients [42]. This group hypothesized that the decrease in dendritic cell activation of the sentinel lymph node led to melanoma development and that, with application of CpG-B, they could immunopotentiate the lymph node by allowing the DNA sequences that bind to TLR-9 to activate dendritic cells. They found that administering the CpG-B vaccine led to higher activation of dendritic cells in the sentinel lymph node and increased the variety of cytokines [42]. There may be a synergistic effect when CpG vaccines are used in combination with other therapies, and the fact that some patients in the Phase II trial with glioblastoma had a long-term response implies there may be some undefined biological or clinical characteristics that make patients more likely to respond to vaccination [39].

In regard to clinical trials combining TLR agonists with radiation therapy, in one study, 15 patients with mycosis fungoids, a subtype of cutaneous T-cell lymphoma, were treated with intratumoral TLR9 agonist and radiation, and distant untreated sites were monitored for systemic responses. Five patients responded to treatment and showed reduced levels of Tregs [43]. From the existing clinical and pre-clinical data, it becomes clear that TLR agonists are a powerful tool in priming radiation as an in situ cancer vaccine.

## 5. Viral Component-Based Vaccines 

Another anti-cancer vaccine strategy are viral vaccines, in which a virus is used to potentiate a patient’s immune system against the tumor. One preclinical study treated a colon cancer animal model expressing carcinoembryonic antigen (CEA) with a combination of radiation therapy and recombinant fowlpox vectors [44]. The group found that the combination of this recombinant vaccine and radiation resulted in better overall survival compared to either treatment plan alone [45].

In terms of clinical trials that combine viral vaccines and radiation, there is currently a Phase I study in which a group of patients were given a combination of IL-2, rV-PSA/rV-B7.1, and radiation. Most of the patients tolerated the treatment plan well and several patients showed an increased number of prostate-specific antigen T cells versus the groups that received radiation therapy alone [46]. However, the US National Cancer Institute that conducted this trial did a long-term follow up after the study to look at, in patients treated with radiation and poxvirus vaccine, overall survival, toxicity, and prostrate-specific antibody numbers. The study found no significant differences in these factors [47]. Regardless, there are more clinical trials underway to show the positive effects of radiation and viral vaccine combinations.

## 6. Protein/Peptide Subunit Vaccines 

Another effective vaccine combination has been that of protein peptide complexes paired with radiation. One pre-clinical group investigated human papillomavirus (HPV)-related head and neck cancer and gave tumor-bearing mice (TC-1 model) a Shiga toxin-based E7 vaccine before and after a radiation dose of 7.5 Gy [48]. The treated mice survival rate was a staggering 70%. In another pre-clinical study with an HPV cervical cancer model, a subcutaneous E7 protein and a CpG-oligodeoxynucleotide subunit vaccine was given in combination with a single high-radiation dose [49]. This particular combination led to tumor regression and long-term immune memory compared to the radiation alone groups. There are currently several ongoing clinical trials involving protein/peptide vaccines with radiation. One such trial involves a vaccine made of isolated protein peptide complex 96, known as Oncophage [50,51], and radiation for the treatment of pediatric patients with glioma (NCT02722512). This same protein vaccine has been used in a Phase II trial (NCT00905060) and was given intradermally after treatment with radiation and temozolomide chemotherapy. Various pre-clinical and clinical studies reveal that the combination of radiation and protein/peptide vaccines is proving effective, but there is still much improvement to be made in transferring these treatment plans into clinic.

## 7. Conclusions

Undoubtedly, progress has been made in the area of cancer vaccines, but there is still much needed investigation to be held. While the anti-cancer vaccines of MUC1, p53, CpG, and SOX2 have some pre-clinical and clinical efficacy, there is an increasing amount of evidence that points to radiation’s effectiveness as an in situ vaccine that can prime the patient’s immune system against systemic disease. The recent pre-clinical and clinical evidence combining radiation with vaccine types such as TLR, viruses, and peptides, provide compelling evidence for synergism that exists between the two therapies. With more studies in the pipeline, it is becoming clear that radiation plays a crucial part in shaping the future of cancer vaccine development.
